# Editorial: Application of Genetically Encoded Indicators to Mammalian Central Nervous System

**DOI:** 10.3389/fnmol.2015.00076

**Published:** 2015-12-22

**Authors:** Yoshiyuki Yamada, Katsuhiko Mikoshiba

**Affiliations:** ^1^Laboratory for Developmental Neurobiology, Brain Science Institute, RIKENWako-shi, Japan; ^2^Central Institute for Experimental AnimalsKawasaki-shi, Japan

**Keywords:** genetically encoded sensors, fluorescent protein sensors, genetically encoded calcium indicators (GECIs), *in vivo* imaging, genetically encoded voltage sensor

Genetically encoded indicators are fluorescent proteins engineered to change their optical properties and report the dynamics of intracellular molecules or voltage. Encoded in a relatively compact size of DNA (typically 2–3 kilobase pairs), they are readily compatible with standard gene delivery techniques, including viral vectors and transgenesis. They can be thus expressed in specific types of cells with the use of cell type-specific promoters and/or Cre-loxP recombination system, which makes them beneficial for disentangling the complex mammalian neural circuitry. Another unique advantage of genetically encoded indicators is that they can be stably expressed for a long period, which allows chronic tracking of the same neuronal ensemble by longitudinal imaging, and offers unprecedented opportunities to investigate the neural basis of development, learning, and disease.

Since the prototypical genetically encoded Ca^2+^ indicators (GECIs) were reported for the first time (Miyawaki et al., 1997), genetically encoded indicators have drastically evolved in terms of signal to noise ratio, response kinetics, and spectral variety. In this Research Topic, Tian and colleagues provide a review on genetically encoded indicators of neuronal activity in general, including GECIs, with a special focus on the GCaMP family (GECIs composed of a circularly permutated GFP, the Ca^2+^-binding protein calmodulin, and the calmodulin-binding peptide M13), as well as genetically encoded indicators of voltage and synaptic activity (Broussard et al.). Griesbeck and colleagues present an overall review of GECIs, covering from the basics of Ca^2+^ imaging in neurons to the description of latest variants, and also discuss recent studies where GECIs were applied to neuronal population imaging in awake head-restrained mice (Rose et al.). The article by Nagai and colleagues focuses on affinity variants, color variants and photoactivatable variants of GECIs, the last two of which are of considerable importance for simultaneous use with optogenetic tools (Nagai et al.). Lin and colleagues then provide a review, for the first time to our knowledge, on a rapidly growing subfamily of optogenetic tools—a group of photoactivatable effector proteins that control the dynamics of intracellular molecules, rather than the membrane voltage (Zhou et al.).

It is also noteworthy that an important update of red GECI, R-CaMP2 was recently published (Inoue et al., 2015), reflecting the highly active nature of this field. R-CaMP2 was shown to have fast kinetics, high linearity, and remarkable sensitivity to detect single action potentials, making it even competitive with the state-of-art green GECIs; the proof of principle experiments show that it can be simultaneously used with GCaMP and/or channelrhodopsin, paving the way to multicolor longitudinal imaging and all-optical manipulation/recording of neural activity. Another group is also developing different variants of red GECIs independently (Dana et al., 2014), and it would be interesting to see side-to-side comparison in the future.

More specific technical advances are also reported in this Research Topic. Knöpfel and colleagues characterize a new series of genetically encoded voltage indicators, with a chimeric voltage sensing-domain (fragments from the Ci-VSP and the Kv3.1 potassium channel) sandwiched by a FRET donor and acceptor (“butterfly” design; Mishina et al.). Tsutsui and colleagues present their attempts to turn a GFP variant into a genetically encoded chromophore optimized for second harmonic generation microscopy, which can be used for voltage imaging (Jinno et al.). White and colleagues describe a set of *in utero* electroporation vectors carrying genetically encoded Ca^2+^ indicators (GCaMP), and investigate Ca^2+^ dyanamics of astrocytes in rat organotypic slice culture (Gee et al.).

The improved performance and rich repertoire of genetically encoded indicators have stimulated their widespread application to biologically relevant questions. Tvrdik and colleagues demonstrate 2-photon Ca^2+^ imaging in transgenic mice expressing GCaMP in microglia, and study Ca^2+^ dynamics induced by lipopolysaccharide and focal laser injury (Pozner et al.). Ohki and colleagues perform wide-field Ca^2+^ imaging in transgenic mice expressing GCaMP broadly across the cortex, and identify two visual association areas that respond to drifting gratings, where they further examine the cellular responses by 2-photon imaging (Murakami et al.). Fletcher and colleagues also perform wide-field Ca^2+^ imaging in transgenic mice expressing GCaMP in excitatory output neurons in the olfactory bulb, and examine the circuit element responsible for olfactory habituation (Ogg et al.).

We thank all the authors and reviewers for their valuable contributions to our Research Topic, and sincerely hope that it will provide readers with useful information and inspiration for further innovation in the field.

## Author contributions

YY and KM conceived the overall plan of Research Topic, recruited contributors, and wrote the manuscript.

### Conflict of interest statement

The authors declare that the research was conducted in the absence of any commercial or financial relationships that could be construed as a potential conflict of interest.
